# A promising metabolite, 9-aminominocycline, restores the sensitivity of tigecycline against *tet*(X4)-positive *Escherichia coli*

**DOI:** 10.3389/fmicb.2024.1432320

**Published:** 2024-07-08

**Authors:** Feifei Sun, Lin Zhang, Xuan Ma, Tariq Ali, Yongning Wu, Lin Li

**Affiliations:** ^1^Animal-Derived Food Safety Innovation Team, College of Animal Science and Technology, Anhui Agricultural University, Hefei, China; ^2^NHC Key Laboratory of Food Safety Risk Assessment, China National Center for Food Safety Risk Assessment, Beijing, China; ^3^College of Veterinary Sciences, University of Agriculture, Peshawar, Peshawar, Pakistan

**Keywords:** 9-aminominocycline, tigecycline, bacterial resistance, Tet(X4) inactivating enzyme, antibacterial adjuvant

## Abstract

The emergence and widespread of tigecycline resistance undoubtedly poses a serious threat to public health globally. The exploration of combination therapies has become preferred antibacterial strategies to alleviate this global burden. In this study, tigecycline-resistant *tet*(X4)-positive *Escherichia coli* were selected for adjuvant screening. Interestingly, 9-aminominocycline (9-AMC), one of the tigecycline metabolites, exhibits synergistic antibacterial activity with tigecycline using checkerboard assay. The efficacy *in vitro* and *in vivo* was evaluated, and the synergistic mechanism was further explored. The results suggested that 9-AMC combined with tigecycline could inhibit the growth of antibiotic resistant bacteria, efficiently retard the evolution of *tet*(X4) gene and narrow the drug mutant selection window. In addition, the combination of tigecycline and 9-AMC could destroy the normal membrane structure of bacteria, inhibit the formation of biofilm, remarkably reduce the level of intracellular ATP level, and accelerate the oxidative damage of bacteria. Furthermore, 9-AMC is more stable in the bind of Tet(X4) inactivating enzyme. The transcriptomics analysis revealed that the genes related to the 9-AMC and tigecycline were mainly enriched in ABC transporters. Collectively, the results reveal the potentiation effects on tigecycline and the probability of 9-AMC as a novel tigecycline adjuvant against *tet*(X4)-positive *Escherichia coli,* which provides new insights for adjuvant screening.

## Introduction

Antibiotics are one of the most transformative drugs in human history, altering the trajectory of human life (Zheng et al., 2022). However, the abuse of antibiotics led to the emergence and widespread dissemination of bacterial resistance, posing a great challenge to clinical treatment and public health (Caneschi et al., 2023; Ren et al., 2023; Ueda et al., 2023). Tigecycline, a third-generation tetracyclines, has good antibacterial activity against Gram-negative, Gram-positive, anaerobic bacteria, and multi-drug-resistant pathogens (Peterson, 2008; Sodeifian et al., 2023). The emergence of the plasmid mediated colistin resistance gene, *mcr*-1, has dramatically reduced the clinical efficacy of colistin, enabling tigecycline the “last line of defense” for the treatment of multidrug-resistant pathogenic bacterial infections (Ruan et al., 2020). With the widespread use of tetracyclines, increasing tigecycline resistance was observed. Tigecycline resistance mechanisms include nonspecific efflux pump action, ribosomal protection, cell membrane permeability, and the tetracycline inactivating enzyme Tet(X) (Chen et al., 2017; He et al., 2019; Wang et al., 2021; Cui et al., 2022). Tet(X), a flavin-dependent monnoxygenase originally discovered on the R plasmid, could hydroxylate tigecycline into 11α-hydroxy tigecycline, thereby weakening the antibacterial activity (Forsberg et al., 2015; Markley and Wencewicz, 2018). In particular, the emergence and widespread of high-level tigecycline resistance mediated by *tet*(X3/X4) in *Escherichia coli* seriously constitutes a threat for public health globally (Wang et al., 2020; Li et al., 2021; Mohsin et al., 2021).

The development of novel antibiotics is undoubtedly an effective way to overcome the growing drug resistance. However, limited by high cost, and long cycle for a new drug development, only several antibiotics with novel skeletons were developed. The combination of β-lactams (penicillins, cephalosporins, and carbapenems) with β-lactamase inhibitors through microbial screening and chemical structure modification of enzyme inhibitors is a typical successful case of combination therapy (Li et al., 2022; Yang et al., 2023). Similarly, the antibiotic adjuvants or identification of inhibitors targeting *tet*(X3/X4) is a potential alternative to enhance the antibacterial efficacy against multidrug resistant strains.

The tigecycline-based combination therapies including adjuvants and inhibitors were by interfering intrinsic resistance mechanisms or enhancing antibiotic killing capability (Dhanda et al., 2023; Hu et al., 2023). The existed combination strategies can be divided into antibiotic and non-antibiotic adjuvants. The metabolites, sharing the same skeleton with the parent drug, have been severely underestimated as potential antibacterial adjuvants. 9-Aminominocycline, a metabolite of tigecycline, has the same parent structure as tigecycline and is expected to act as an antimicrobial potentiator for reversing tigecycline resistance and enhancing the antimicrobial effect of tigecycline. Interestingly, our studies revealed that 9-AMC, could remarkably potentiate the antibacterial activity of tigecycline against *tet*(X4) in *Escherichia coli*, and was characterized as a potential adjuvant of tigecycline. However, the mechanism of 9-AMC and tigecycline displaying synergistic activity remains further exploration.

**Figure 1:**
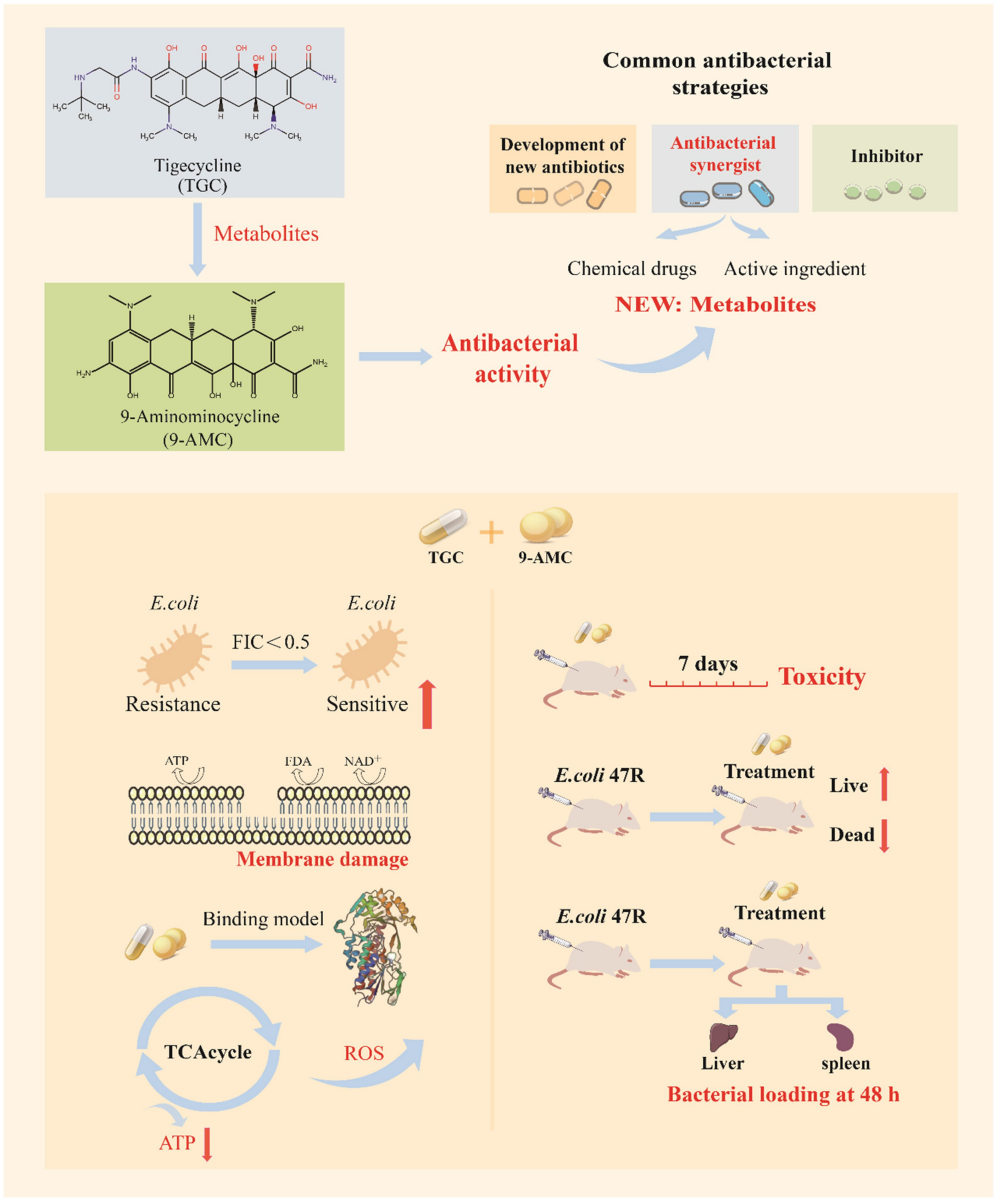
The schematic diagram of 9-AMC as a novel adjuvant of tigecycline against *tet*(X4)*-*positive *E.coli.*

Herein, the *in vitro* combination efficacy of 9-AMC and tigecycline was evaluated by checkerboard assay, time-bactericidal curve and growth curve, and the *in vivo* antimicrobial activity and safety evaluation were confirmed using the animal infection models. To clarify the effects of the combination on the evolution of drug-resistant genes, a resistance transmission assay and the determination of the mutant prevention concentration (MPC) were carried out. Furthermore, transcriptome, scanning electron microscopy, biofilm inhibition and scavenging assay, membrane permeability assay, ATP level and reactive oxygen species assay, proton motive force assay, trans-membrane potential assay, and molecular docking were conducted to reveal the synergistic antimicrobial mechanism of 9-AMC and tigecycline (Figure 1). Conjointly, the obtained results will provide new ideas for the screening of antimicrobial synergists and new solutions for the prevention and control of bacterial resistance.

## Materials and methods

### Bacteria and chemical reagents

The bacteria, *tet*(X4)-positive *E.coli* used in the current study were described in detail in Supplementary Table S1. Briefly, *E.coli* 47R and *E.coli* 2DZ50T were from the pig farms and pig slaughterhouses in Qingdao. The strains were cultured in Luria Bertani broth (LB, Hopebio, Qingdao). Tigecycline and 9-AMC were purchased from Dalian Meilun Biotechnology Co., Ltd. and Shanghai ZZBio Co., Ltd., respectively. The other antibiotics used in the current work were from Shanghai yuanye Biotechnology Co., Ltd. The enhanced ATP assay kit (S0027) and reactive oxygen species assay kit (ROS, S0033S) were obtained from Beyotime Biotechnology Co., Ltd. The fluorescent probes, 1-N-phenylnaphthylamine (NPN), propidium iodide (PI) and DIOC2(3) were purchased from Shanghai Macklin Biotechnology Co., Ltd., while the BCECF-AM from Shanghai Biyuntian Biotechnology Co., Ltd., ethidium bromide (EtBr) and the inhibitor CCCP from Shanghai Aladdin Biochemical technology Co., Ltd. ICR lines, SPF-grade mice, bedding and sterile feed were purchased from Spearfish(Beijing) Biotechnology Co., Ltd.

### Construction and expression of Tet(X4)

The *tet*(X4) genes were obtained from *tet*(X4)-positive *E.coli* published in NCBI (NCBI reference sequence: NG_065852.1). The gene was cloned into the expression vector pET30a(+) with the restriction endonucleases on the cleavage sites of *Nde*I and *Hind*III. The recombinant plasmid pET30a(+) were transferred into *E.coli* DH5α receptor cells to obtain the *E.coli* DH5α-pET30a + *tet*(X4). The expression of Tet(X4) was realized through the prokaryotic system. The primers were described in Supplementary Table S2.

### Antimicrobial susceptibility assay

The minimal inhibitory concentration (MIC) determination for all compounds were conducted abiding by the Clinical and Laboratory Standard Institute (CLSI) guideline. Briefly, the candidate compounds were 2-fold diluted in the CAMHB in a sterile 96-well plate, and 100 μL of bacterial suspensions (1×10^6^ colony forming unites, CFUs/mL) were added into each well. After 16–20 h incubation at 37°C, the MICs of tigecycline and its metabolites on the four strains [*E.coli* ATCC 25922, *E.coli* 47R, *E.coli* 2DZ50T and *E.coli* DH5α-pET30a + *tet*(X4) strains] were determined by microbroth dilution method, where *E.coli* ATCC 25922 was quality control strain.

### Checkerboard assay

The synergistic antibacterial activity between tigecycline and 9-AMC against *tet*(X4)-positive strains was evaluated by the checkerboard micro-dilution assay. Concisely, 100 μL of bacterial suspensions (1×10^6^ CFUs/mL) was added into 96-well plate. The compound candidates starting from MIC were 2-fold serially diluted in CAMHB. The overnight culture was adjusted to a 0.5 McFarland turbidity standard, followed by 100-fold dilution in CAMHB. The MIC values of each combination were monitored, and the fractional inhibitory concentration index (FICI) was calculated according to the formula 1. The synergy effect is defined as an FIC index of ≤0.5.

(1) $$\begin{array}{l} \mathrm{FICI}=\left(\mathrm{MIC}\;\mathrm{of}\;\mathrm{compounds}\;\mathrm{in}\;\mathrm{combination}/\mathrm{MIC}\;\mathrm{of}\;9-AMC\;\mathrm{alone}\right)\\ \;+\left(\mathrm{MIC}\;\mathrm{of}\;\mathrm{tigecycline}\;\mathrm{in}\;\mathrm{combination}/\mathrm{MIC}\;\mathrm{of}\;\mathrm{tigecycline}\;\mathrm{alone}\right)\text{.} \end{array}$$

### Time-dependent killing curve

The overnight culture of *E.coli* 47R, *E.coli* 2DZ50T and *E.coli* DH5α-pET30a + *tet*(X4) was 100-fold diluted before being added into 10 mL of fresh LB media. The culture was treated with PBS, tigecycline alone (1 μg/mL), 9-AMC alone (32 μg/mL), or tigecycline and 9-AMC in combination (1 μg/mL tigecycline+32 μg/mL 9-AMC). Ten μL of bacterial suspension was sequentially collected after incubation at 0, 2, 4, 6, 8, 10, 12, and 24 h, followed by serially dilution before coated on LB agar. After overnight incubation at 37°C, the colony counts were determined to perform the time-dependent killing curves.

### Growth curve

The tested strains were cultured in LB broth to assess the effects of drug combination on the bacterial growth. The overnight culture was adjusted to OD_600nm_ = 0.3, and then diluted 100 times before adding 9-AMC at different concentrations ranging from 2 to 32 μg/mL. The mixed culture was co-incubated at 37°C, and the absorbance at different time points (2, 4, 6, 8, 10, 12, 14, 16, 18, 20, 22, and 24 h) at 600 nm was monitored to plot the growth curves.

### Resistance study

The *E.coli* DH5α-pET30a + *tet*(X4) was cultured in the presence of tigecycline (0.5 MIC), or 9-AMC (0.25 MIC), or the combination (0.5 MIC tigecycline +0.25 MIC 9-AMC). After incubation at 37°C for 12 h, and the bacterial suspension was 100-fold diluted before being cultivated on the new plate for 24 passages. The MIC was determined every four passages by broth micro-dilution assay.

### Mutation preventive concentration determination

The strain, *E.coli* DH5α-pET30a + *tet*(X4), was incubated in MH broth overnight. The bacterial suspension was centrifuged and re-suspended in 200 mL of fresh MH broth. The bacterial suspension was adjusted to 6 ×10^9^ CFU/mL. Then, a total of 100 μL of inoculum was streaked onto agar plate with tigecycline alone, 9-AMC alone or in combination at the same concentration. The mutant prevention concentration (MPC) was defined as the minimum concentration that limited the growth of the bacteria after incubation for 72 h at 37°C.

### Scanning electron microscope analysis (SEM)

The bacteria *E.coli* DH5α-pET30a + *tet*(X4) was overnight cultured in 10 mL of broth media, and treated with PBS, tigecycline, 9-AMC or their combination. Then the bacterial suspension was centrifuged at 4,000 rpm for 5 min, and precipitate was collected and re-suspended in PBS. The samples were fixed and dehydrated, followed by gold spraying before observed using SEM.

### Biofilm inhibition and removal assays

The overnight cultured *E.coli* DH5α-pET30a + *tet*(X4) suspension was 100-fold diluted and added into LB broth with tigecycline alone (0.25–2 μg/mL), 9-AMC (16 μg/mL), or in combination. After 48 h static incubation at 37°C, the bacteria were washed three times using 300 μL of sterile PBS. Then, 200 μL of methanol and 200 μL of 0.1% crystal violet were subsequently used to fix and stain the bacteria. Finally, 200 μL of 33% acetic acid was used to dissolve the biofilm, and incubated at 37°C for 30 min. The biofilm formation in the presence of different drug concentrations was determined by measuring the absorbance of acetic acid at 570 nm. The number of colonies in each group after being cultured for 18 h at 37°C were counted to evaluate the effects of different groups on the biofilm removal.

### Effects on membrane permeability

*E. coli* DH5α-pET30a + *tet*(X4) was incubated with the speed of 180 rpm at 37°C to exponential phase; and centrifuged for 10 min at 5,000 r/min. The bacterial suspensions were collected, where the probe NPN and PI was individually added to a final concentration of 10 μmol/mL and 15 μmol/mL, to evaluate the effects of drug combination on the extracellular and intracellular membrane permeability, respectively. For samples added with NPN, the fluorescence intensity was measured at an excitation wavelength of 350 nm and an emission wavelength of 420 nm. For samples added with PI, the fluorescence intensity was measured with an excitation wavelength of 535 nm and an emission wavelength of 615 nm.

### Proton motive force assay

The proton motive force was evaluated by measuring the intra-and extracellular pH gradient (ΔpH) using the pH-sensitive fluorescence probe BCECF-AM and cellular trans-membrane potential (ΔΨ) using the fluorescence probe DIOC2(3). The bacterial suspension was treated with tigecycline (1 μg/mL), 9-AMC (4–32 μg/mL), or in combination, and the fluorescence intensity was monitored.

### Efflux pump activity

The activity of efflux pump was evaluated using the fluorescent probe ethidium bromide (EtBr) with the final concentration of 5 μmol/mL. The bacterial suspension was treated with CCCP (control group), tigecycline (1 μg/mL), 9-AMC (4–32 μg/mL), or in combination. The fluorescence intensity was measured with an excitation wavelength of 530 nm and an emission wavelength of 600 nm.

### ATP determination

*E. coli* DH5α-pET30a + *tet*(X4) was incubated to exponential phase and centrifuged for 10 min at 4000 r/min. The bacterial suspension was treated with tigecycline (1 μg/mL), 9-AMC (4–32 μg/mL), or in combination. Finally, the suspension was centrifuged at 4°C for 5 min at 12,000*g*, and the supernatant was discarded. The level of ATP was detected by Enhanced ATP Assay Kit.

### Reactive oxygen species assay

*E. coli* DH5α-pET30a + *tet*(X4) was incubated to exponential phase and centrifuged for 10 min at 4,000 r/min. Then, the bacterial suspension was collected and the probe, 2′7′-dichlorodihydrofluorescein diacetate (DCFH-DA) was 1,000-time diluted and added and incubated for 1 h, followed by treatment with tigecycline (1 μg/mL), 9-AMC (4–32 μg/mL), or in combination. The fluorescence intensity was measured using a multifunctional enzyme labeling instrument at an excitation wavelength of 488 nm and an emission wavelength of 525 nm.

### Molecular docking

Tigecycline and 9-AMC were imported into Chem3D software to obtain their 3D chemical structures with the minimum energy. The 3D crystal structure of Tet(X4) was retrieved from Protein Data Bank (PDB ID: 7EPV) and imported into Autodock 4.2 software for charge and structure optimization. The optimal conformations were selected for visualization of the binding sites by Pymol to identify the key amino acids and interaction forces between the target protein and different molecules.

### Transcriptomics analysis

The overnight culture of the tested bacteria was diluted and incubated to OD_600nm_ of 0.5, and treated with tigecycline (1 μg/mL), or in combination (1 + 32 μg/mL). After incubation for 8 h, the total RNA was extracted using RNAprep pure Bacteria Kit. The RNA was quantified using NanoDrop spectrophotometers. The qualified RNA was analyzed by Shanghai Ouyi Biomedical Technology Co., Ltd. The results of transcriptome sequencing were verified by RT-PCR using the internal reference gene 16S rRNA in *E. coli* as a reference. The experiments were performed in triplicates.

### Acute toxicity

Eighteen ICR line, SPF grade mice were randomly divided into 3 groups (*n* = 6 per group) after acclimatization. Then each group was treated with 200 μL PBS, tigecycline (20 mg/kg body weight), or in combination (20 mg/kg body weight for tigecycline and 128 mg/kg body weight for 9-AMC) through intraperitoneal administration. The mice were carefully observed for seven consecutive days and the body weight as well as the biochemical indices were measured and recorded.

### Survival test

Thirty-two ICR line, SPF grade mice were randomly divided into 4 groups (*n* = 8 per group) after acclimatization. Then each group was injected intraperitoneally with 200 μL of 1×10^8^ CFU of *E.coli* 47R bacterial suspension, followed by treatment with 100 μL of PBS, tigecycline (8 mg/kg), 9-AMC (5 mg/mg), and tigecycline+9-AMC (8 + 5 mg/kg), respectively. The mice were observed for 7 consecutive days and the survival of each group was recorded.

### Mouse peritonitis infection model

Thirty ICR line, SPF grade mice were randomly divided into 5 groups (*n* = 6 per group) after acclimatization. Then each group was injected intraperitoneally with 100 μL of 1×10^7^ CFU of *E.coli* 47R bacterial suspensions. After 1 h infection, the tested animals were treated with 100 μL PBS, tigecycline (8 mg/kg), 9-AMC (5 mg/kg), tigecycline +9-AMC (8 + 5 mg/kg) or tigecycline +9-AMC (20 + 5 mg/kg), respectively. At 48 h post-infection, the blood was collected from the orbits to determine the inflammatory factors (IL-1β, IL-6, TNF-α). The mice were euthanized through cervical dislocation, and the duodenum was collected to compare the histopathological changes in each group. The spleen and liver of the mice were aseptically removed and homogenized, diluted before incubation in the LB agar plate. The bacterial numbers were counted after incubation 37°C for 18–20 h.

### Ethics statement

All the animal experiments were approved by the Animal Welfare and Ethics Committee at Anhui Agricultural University with the approval ID of AHAUXMSQ2023052.

### Statistical analysis

The statistical analysis was conducted using GraphPad Prism 8.0. The data were described as means ± SD. The ANOVA or *t*-test analysis were performed to calculate *p*-values (*p < 0.05, **p < 0.01, ***p < 0.001, ****p < 0.0001).

## Results

### 9-AMC exerts an excellent synergistic activity with tigecycline

**Figure 2:**
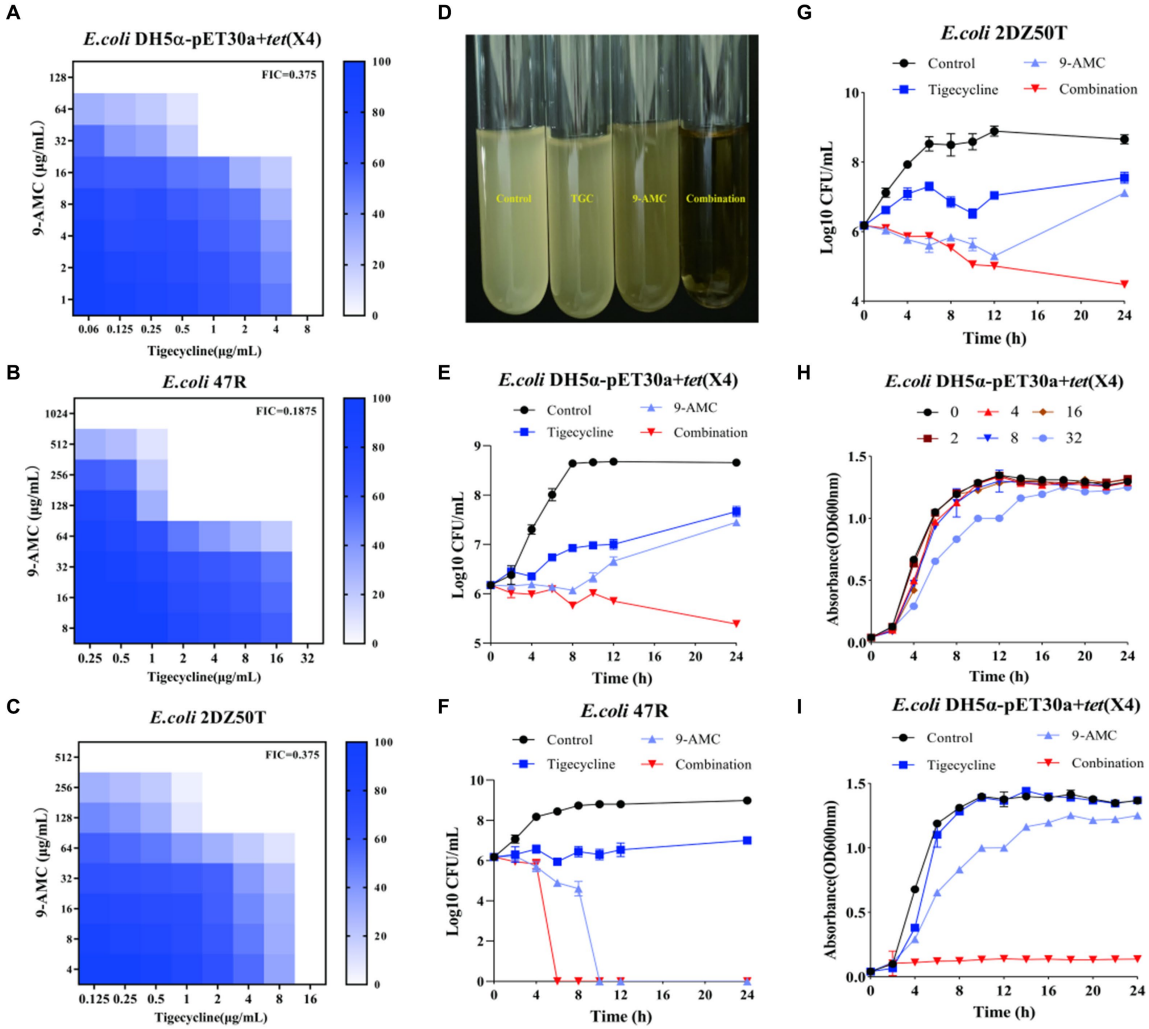
9-AMC restored the antibacterial activity of tigecycline *in vitro* against the *tet*(X4)-positive *E.coli.* FICI values for *E.coli* DH5α-pET30a + *tet*(X4) **(A)**; *E.coli* 47R **(B)**; *E.coli* 2DZ50T **(C)**. The combination of 9-AMC and tigecycline led to bacterial lysis **(D)**. Time-dependent killing curves of *E.coli* DH5α-pET30a + *tet*(X4) **(E)**, *E.coli* 47R **(F)**, and *E.coli* 2DZ50T **(G)**. The growth curve of *E.coli* DH5α-pET30a + *tet*(X4) exposed to different concentrations of 9-AMC **(H)**. The growth curve of *E.coli* DH5α-pET30a + *tet*(X4) treated with 9-AMC, tigecycline alone, or in combination **(I)**.

9-AMC, as one of the metabolites of tigecycline, showed synergistic antibacterial activity *in vitro* and *in vivo*, the chemical structure and the mass spectrum of 9-AMC were provided in Supplementary Figure S1. To explore the potential antibacterial synergists against *tet*(X4)-positive bacteria including *E.coli* DH5α-pET30a + *tet*(X4) and two clinical isolates (*E.coli* 47R and *E.coli* 2DZ50T), the MICs of tigecycline and 9-AMC against the three strains were determined by microbroth dilution. All of the tested strains were determined to be resistant to tigecycline (MIC ≥8 μg/mL) according to CLSI. The MIC values of tigecycline were 267-1067-fold higher against the three *E. coli* strains compared to the sensitive strain *E. coli* ATCC 25922. In addition, 9-AMC exhibited *in vitro* antimicrobial activity against all tigecycline-resistant strains, inhibiting bacteria at high concentrations (128–1,024 μg/mL). The synergistic effects of 9-AMC and tigecycline were investigated using the checkerboard method and the FICI values were calculated. The main strains tested were *E.coli* DH5α-pET30a + *tet*(X4), *E.coli* 47R and *E.coli* 2DZ50T, and the corresponding FICI values were 0.375 for *E.coli* DH5α-pET30a + *tet*(X4) (Figure 2A), 0.1875 for *E.coli* 47R (Figure 2B), and 0.375 for *E.coli* 2DZ50T (Figure 2C), respectively, indicating the synergistic antimicrobial effects. Generally, 9-AMC enhanced the susceptibility of tigecycline against *tet*(X4)-positive *E.coli* by 8–16 folds. As illustrated in Figure 2D, the bacterial CFUs/mL at different time points during 24 h treated with PBS, tigecycline, or 9-AMC, or in combination indicated that the combination treatment led to bacterial lysis. The combination of 9-AMC and tigecycline exhibited obvious bactericidal activities against *E.coli* DH5α-pET30a + *tet*(X4) (Figure 2E), *E.coli* 47R (Figure 2F), and *E.coli* 2DZ50T (Figure 2G). The *E.coli* DH5α-pET30a + *tet*(X4) was used to explore the effect of 9-AMC on the bacterial growth. In Figure 2H, 9-AMC did not significantly inhibit the growth of *E.coli* DH5α-pET30a + *tet*(X4) when used alone within 24 h. Notably, tigecycline alone at the concentration of 1 μg/mL could not affect the bacterial growth (Figure 2I), but when tigecycline was used at the same concentration (1 μg/mL) in combination with 9-AMC, a significant inhibitory effect on the growth of bacteria was observed.

### 9-AMC minimizes the emergence of tigecycline resistance

**Figure 3:**
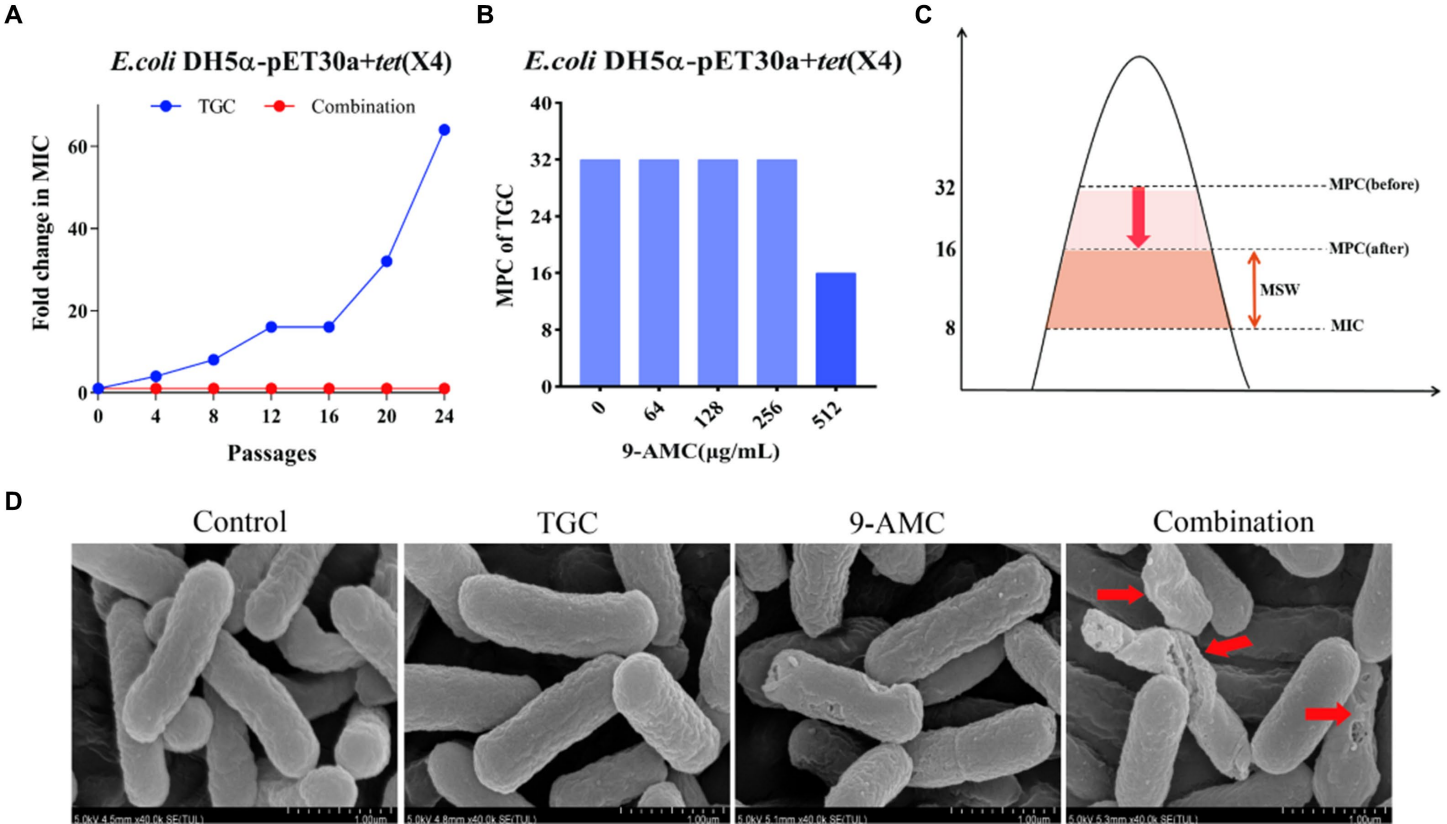
9-AMC can hinder the evolution of drug resistance genes and reduce the MSW of tigecycline. After 24th passages, the MIC of the combination group did not increase, while the MIC of the monotherapy group increased by 64 **(A)**. The MPC value of tigecycline for *E.coli* DH5α-pET30a + *tet*(X4) is 32 μg/mL. High concentrations of 9-AMC(512 μg/mL) can reduce the MPC value of tigecycline by half **(B)**, reducing MSW **(C)**. The morphological changes of the tested bacteria treated with mono-therapy or combination therapy **(D)**.

To better understand the influence of 9-AMC on the development of tigecycline resistance, 24 passages of the tested bacteria, *E.coli* DH5α-pET30a + *tet*(X4), with sub-MIC of tigecycline in the presence and absence of 9-AMC were performed. Notably, the MIC value of the tigecycline alone group increased 64-fold, and no resistant mutant was observed in the combination group (Figure 3A). The results suggested that 9-AMC could slow down the tigecycline resistance. To clarify the optimal combination therapy, the mutant selection window (MSW) was explored. MSW refers to the concentration range between the MIC and MPC, where antimicrobial drug within the MSW can promote the emergence and selection of bacterial resistance mutations. Therefore, it should be noted that the antimicrobial drug should avoid the MSW range. In order to investigate whether the presence of 9-AMC could decrease the MPC value of tigecycline and narrow the MSW, the MPC values of tigecycline alone and in combination with different concentrations of 9-AMC were determined. The provisional MPC of tigecycline was measured to be 32 μg/mL, and the MPC was determined to be 32 μg/mL after linearly decreasing the concentration by 20%. Seen from Figure 3B, tigecycline alone has a large MSW, and low concentrations of 9-AMC do not significantly reduce the MPC of tigecycline, but when tigecycline is used in combination with a high concentration of 9-AMC, the MPC can be reduced to 1/2 of its original size, resulting in a narrower MSW (Figure 3C).

### 9-AMC and tigecycline damaged the integrity of the structure of the tested bacteria

The changes in the morphology of the tested bacteria after treatment with PBS, mono-therapy or combination therapy were observed by scanning electron microscopy. As illustrated in Figure 3D, the *E.coli* showed normal morphology of *E. coli* after treatment with PBS or tigecycline alone. However, 9-AMC alone slightly altered the morphology of the tested bacteria. While 9-AMC in combination with tigecycline could significantly change the morphology where the bacteria began to twist and deform, and even rupture and fracture occurred. Thus, the combination therapy exerts the antimicrobial activity by breaking the cell membrane and increasing the cell permeability.

### Effects of 9-AMC and tigecycline on the bacterial membrane

**Figure 4:**
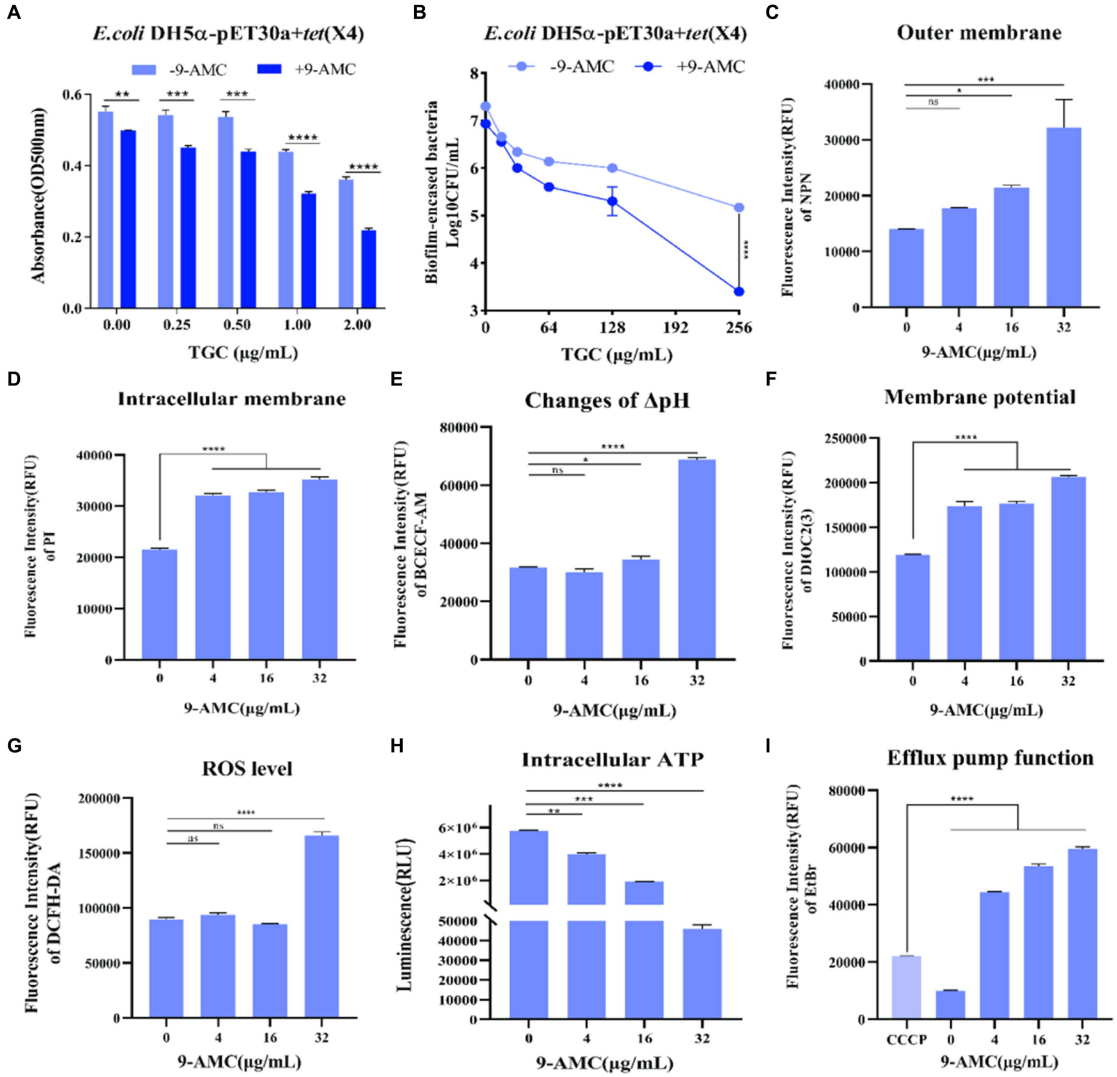
9-AMC can disrupt bacterial membrane structure, affect bacterial energy metabolism, and inhibit efflux pump activity. Compared with the tigecycline alone (0.25–2 μg/mL), the combination of 9-AMC (16 μg/mL) inhibits the normal growth of biofilms, and this inhibitory effect increases with the increase of tigecycline concentration **(A)**. The combination of high concentration tigecycline (256 μg/mL) and 9-AMC (32 μg/mL) can significantly enhance the clearance effect on bacterial biofilm (*p* < 0.0001) **(B)**. Compared with tigecycline alone, the addition of 9-AMC can disrupt the outer **(C)** and inner **(D)** membrane structures of bacteria, affect proton motive force **(E)**, and dissipate membrane potential **(F)**. When tigecycline is used alone (1 μg/mL) or in combination with low concentrations of 9-AMC (4–16 μg/mL), there is no significant difference in intracellular reactive oxygen species content, but when tigecycline is used with 32 μg/mL 9-AMC, intracellular ROS levels significantly increased, and bacteria released a large amount of reactive oxygen species **(G)**. When 9-AMC combined with tigecycline, the ATP synthesis process in the cell is affected, and the intracellular ATP level significantly decreases, and the ATP synthesis level decreases with the increase of 9-AMC concentration **(H)**. The activity of the bacterial efflux pump cannot be inhibited at a low level by tigecycline, but when combined with 9-AMC, it can inhibit the activity of the efflux pump, and this inhibition is stronger than that of the inhibitor CCCP **(I)**. (*p < 0.05, **p < 0.01, ***p < 0.001, ****p < 0.0001).

Interestingly, compared with mono-therapy group, combination therapy remarkably enhanced the inhibitory effect on the biofilm formation process (Figure 4A) and prominently enhanced the removal of mature biofilms of drug-resistant bacteria (Figure 4B). Furthermore, the fluorescent probes, NPN and PI were used to evaluate the extracellular and intracellular membrane permeability by monitoring the fluorescence intensity. As shown in Figure 4C, there was no significant change in fluorescence intensity after the combination therapy of tigecycline and 9-AMC (4 μg/mL). However, the fluorescence intensity was significantly enhanced when the concentration of 9-AMC increased, which indicated that the combination of tigecycline and 9-AMC (16–32 μg/mL) could synergistically disrupt the bacterial extracellular membranes and increase the permeability of bacterial outer membranes. The fluorescence intensity for the combination therapy at various concentrations of 9-AMC increased significantly, indicating that 9-AMC and tigecycline facilitate the damage of inner membrane (Figure 4D), and promote the entry of the drugs into the bacterial, thereby accelerating the rupture and death of the bacteria. The fluorescent probe BCECF-AM and DIOC2(3) were selected to assess the influence of combination therapy on proton motive force (Figure 4E) and trans-membrane potential (ΔΨ) (Figure 4F). The increased fluorescence intensity revealed that the membrane potential and proton motive force changed significantly, which reflected that the combination therapy could disrupted the electric potential, leading to disruption of homeostasis. The membrane potential increases indicating membrane depolarization, which is related to the expression of reactive oxygen species (ROS). To further explore the role of ROS, the fluorescent probe, DCFH-DA was used, and the ROS level treated with tigecycline in presence of 9-AMC at the concentration of 32 μg/mL was greatly increased (Figure 4G), demonstrating the critical role of ROS generation by combination therapy in reversing the resistance. PMF is the driving force for ATP production. The results implied that the combination therapy can significantly decrease the intracellular ATP levels in a concentration-dependent manner (Figure 4H). Since the bacterial efflux pump is correlated with the ATP synthesis, the probe, ethidium bromide was used to explore the effects of 9-AMC on the efflux pump function. The results in Figure 4I revealed that the presence of 9-AMC remarkably inhibited the bacterial efflux pumps.

### Transcription analysis and molecular docking

**Figure 5:**
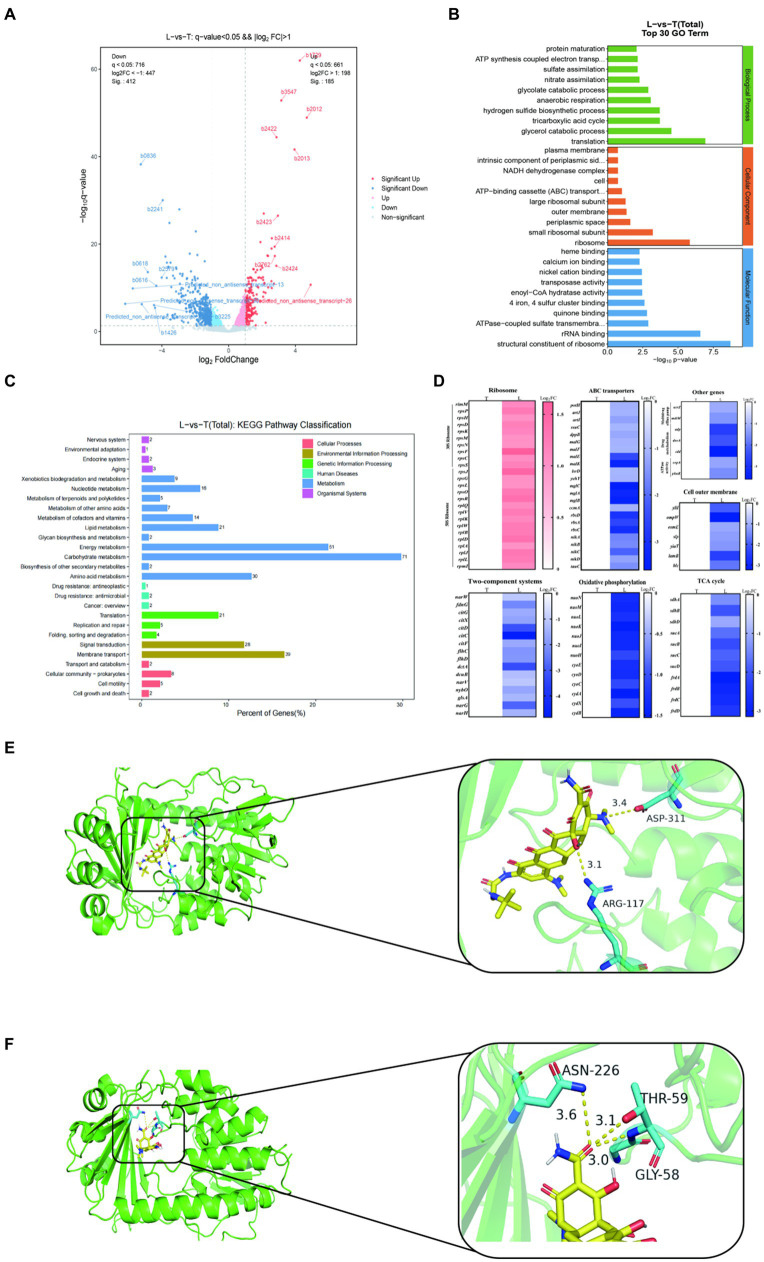
Transcriptome analysis of *E.coli* DH5α-pET30a + *tet*(X4) treated with tigecycline alone (T) or the combination of tigecycline plus 9-AMC (L). Volcano plot **(A)**. GO (gene ontology) annotation analysis of the differential expression genes (DEGs) after exposing tigecycline (1 μg/mL) or the combination of tigecycline (1 μg/mL) plus 9-AMC (32 μg/mL) for 8 h **(B)**. KEGG annotation analysis of DEGs in *E.coli* DH5α-pET30a + *tet*(X4) **(C)**. Selected differential expression genes involved in ribosome, ABC transporters, Two-component systems, oxidative phosphorylation, cell outer membrane, TCA cycle, multidrug efflux pump, drug metabolism and ATPase activity **(D)**. Molecular Docking Analysis of tigecycline **(E)** and 9-AMC **(F)** with Tet(X4) protein, respectively.

To deep understanding the molecular mechanism of 9-AMC and tigecycline to exhibit synergistic antibacterial activity, transcription analysis and molecular docking were carried out. As results, a total of 597 differentially expressed genes (DEG) were screened in the combination group (L) while the tigecycline monotherapy group (T) as a control, of which 185 differentially expressed genes were up-regulated and 412 differentially expressed genes were down-regulated (Figure 5A). GO annotation analysis (Figure 5B), indicated that the differentially expressed genes were correlated with cellular biological processes (translation, metabolism, etc.), cellular components (ribosomes, periplasmic space, extracellular membrane, etc.), and molecular functions (ribosome, rRNA binding, etc.). KEGG enrichment analysis revealed that the DEGs with up-regulated expression mainly enriched in bacterial ribosomes and metabolic pathways (Supplementary Figure S2A) and down-regulated DEGs enriched in cellular metabolic pathways, such as oxidative phosphorylation, pyruvate metabolism, TCA cycle, methyl butyrate metabolism, and glycolate and dicarboxylic acid metabolism, etc. (Supplementary Figure S2B). In general, the DEGs were mainly associated with carbohydrate metabolism, energy metabolism and membrane transport (Figure 5C and Supplementary Figure S2C). Additionally, 30S and 50S ribosome-related genes are up-regulated after treatment with combination therapy compared with mono-therapy (Figure 5D). The significant decrease in the expression of genes related to bacterial ABC transporter and multidrug efflux pump indicated that the addition of 9-AMC could diminish the efflux function of the bacteria, and this diminishing effect might be originated from the down-regulation of the expression of genes related to the upstream regulatory system, the two-component system. The down-regulation of the expression of genes related to the TCA cycle, the process of oxidative phosphorylation, the activity of ATPase. The down-regulation of the expression of genes related to drug metabolism suggests that 9-AMC can affect the bacterial metabolic pathway, which corresponds to the significant decrease in intracellular ATP level and dissipation of membrane potential in the previous ATP assay. Meanwhile, the suppression of outer membrane gene expression also demonstrated that 9-AMC could disrupt the integrity of the bacterial outer membrane and rupture the bacterium, facilitating the entry of the drug into the bacterium. To ensure the reliability, the efflux pump genes (*acrZ*, *mdtM*), extracellular membrane gene (*ompW*), and the ABC transporter genes (*nikA*, *artJ*) were selected to perform fluorescence quantitative PCR verification. The primer sequences are shown in Supplementary Table S3. According to Supplementary Figure S2D, the expression of *acrZ*, *mdtM*, *ompW*, *nikA*, *artJ* genes in the combination group of tigecycline and 9-AMC significantly decreased compared with tigecycline mono-therapy group, which was consistent with the transcriptomics analysis.

The binding mode of 9-AMC and Tet(X4)-inactivating enzyme was determined through molecular docking. Both tigecycline (Figure 5E) and 9-AMC (Figure 5F) were able to bind to the active center of Tet(X4) and, in contrast to tigecycline, 9-AMC also interacted with the other active centers of the FAD of Tet(X4). In addition, the binding energies of 9-AMC and tigecycline with Tet(X4) are −8.27 and −6.23 kJ/mol, respectively, suggesting that 9-AMC binds more stably to Tet(X4). The detailed information for molecular docking was provided in Supplementary Table S4. Furthermore, the mechanism of action for the restoration of the sensitivity of the strain to tigecycline after the addition of 9-AMC was explained from the viewpoint of conformational relationship.

### AMC restore tigecycline resistance *in vivo* and safety evaluation

**Figure 6:**
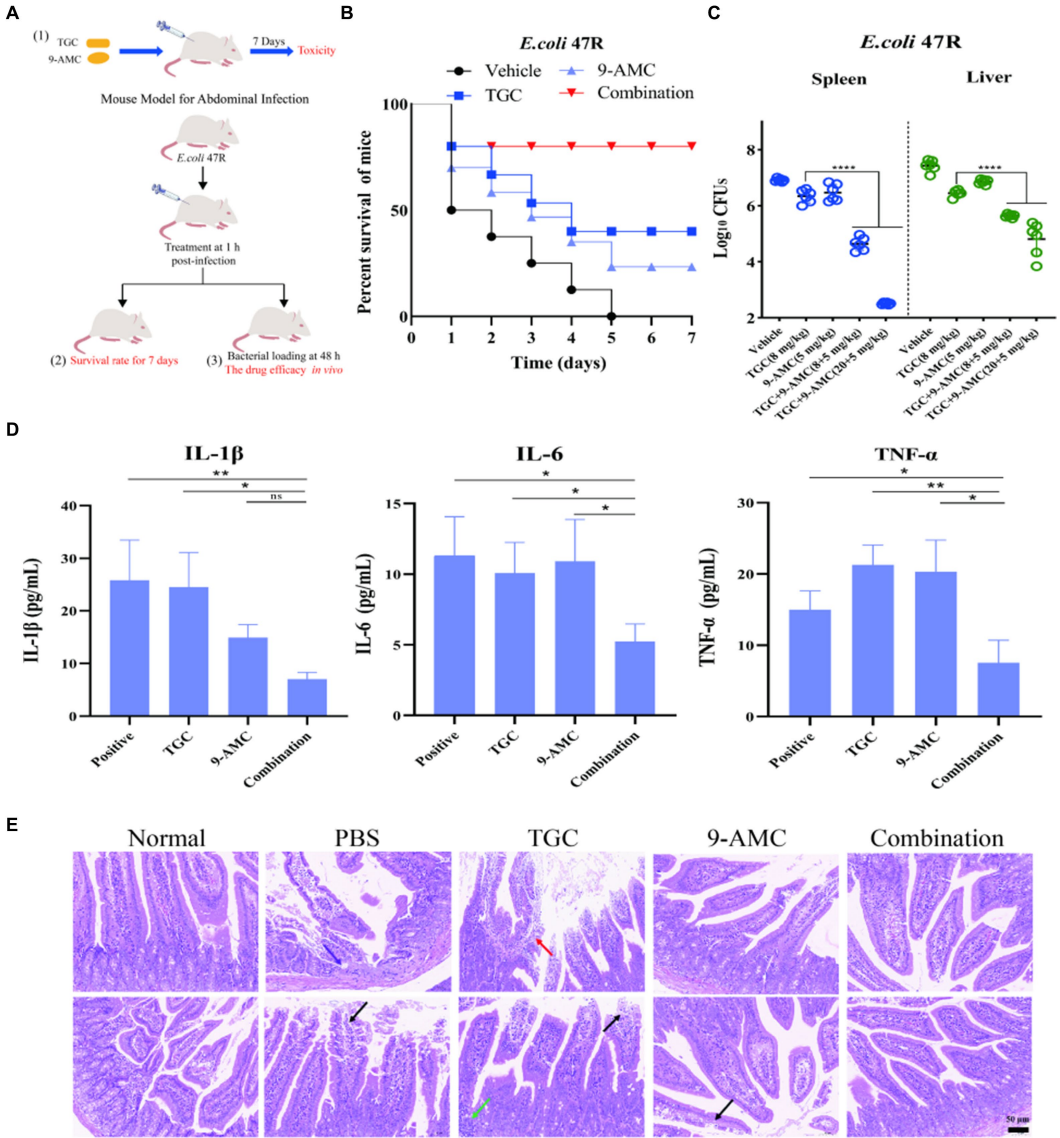
9-AMC can improve therapeutic efficacy, reduce mouse mortality, and alleviate inflammation. The schematic diagram of in vivo safety evaluation for 9-AMC with tigecycline against *tet*(X4)-positive *E.coli* infection **(A)**. Survival rates of mouse (*n* = 8 per group) infected by tigecycline-resistant *E. coli* 47R treated with tigecycline (8 mg/kg) and 9-AMC (5 mg/kg) alone or their combination **(B)**. Bacterial load in liver and spleen infected model after treated with combination therapy significantly decreased compared with the mono-therapy group **(C)**. The levels of IL-1β, IL-6 and TNF-α in serum **(D)**. One-way ANOVA was used to determine statistical significance (ns *p* > 0.05, *p < 0.05, **p < 0.01). The histopathological changes of duodenum treated by mono-therapy or combination therapy **(E)**, where arrows show granulocyte infiltration (purple), losing of mucosal epithelium and exposure of lamina propria (black), lymphocyte and granulocyte infiltration (red) and lymphocytes aggregate into clusters (green).

The potentiation of 9-AMC with tigecycline against *tet*(X4)-positive *E.coli* has been elucidated *in vivo* (Figure 6A). The toxicity of combination therapy at a higher dose (20 mg/kg b.w. for tigecycline and 128 mg/kg b.w. for 9-AMC) were assessed. The body weight (Supplementary Figure S3), blood routine analysis (Supplementary Table S5) and the biochemical indexes (Supplementary Table S6) showed no obvious difference between the combination group and control group, indicating that the combination of 9-AMC and tigecycline within the concentration of 20 mg/kg b.w. for tigecycline and 128 mg/kg b.w. for 9-AMC displayed no toxicity.

The *in vivo* efficacy of the combination therapy was tested in the mouse peritonitis infection model infected with clinical isolate, *E.coli* 47R. As illustrated in Figure 6B, the survival rates of mouse in the vehicle group, tigecycline group, 9-AMC group and in combination group were 0, 37.5, 25, 75%, respectively, suggesting that 9-AMC and tigecycline could significantly potentiate the efficacy against *tet*(X4)-positive *E.coli* infection. It should be noted that, in comparison with the vehicle group or with mono-therapy, the bacterial load decreased slightly. Furthermore, the bacterial load in the combination group treated with tigecycline and 9-AMC at 8 + 5 mg/kg, decreased approximately 1-2-log_10_CFUs in spleen or liver (Figure 6C). The combination therapy with higher concentration (20 + 5 mg/kg) showed more reduced CFUs (2-3-log_10_). The *in vivo* results demonstrated the adjuvant potential of 9-AMC to restore the sensitivity of drug-resistant strains to tigecycline and improve the therapeutic effects. Notably, the combination therapy significantly decreased the levels the inflammatory factors in serum including IL-1β, IL-6, and TNF-α (Figure 6D).

In consistent with the above results, the combination of 9-AMC and tigecycline could alleviate or relieve the histopathological injury. According to the “International Norms for the Terminology and Diagnostic Criteria of Pathological Changes in Rats and Mice (INHAND),” mouse small intestine pathological sections inn different groups were evaluated, and the results were available in Supplementary Table S7. As illustrated in Figure 6E, the normal small intestine of mice in overall and enlargement of partial area where the intestinal villi covered, and a single layer of columnar epithelium, epithelial cells between the distribution of cup-shaped cells, and the Li′s crypts were observed in the negative control group. After infection with *E.coli* 47R, the lesions in the intestinal tissues were observed with small focal erosions in the mucosal layer of intestinal tissues, loss of mucosal epithelium and intestinal glandular structures with granulocyte infiltration (purple arrowheads), and a small amount of mucosal epithelium loss and exposed lamina propria (black arrowheads). The mucosal epithelium of intestinal tissue treated with tigecycline mono-therapy is missing, lamina propria is bare (black arrowheads) and lymphocytes are aggregated into a cluster (green arrowheads). Besides, lymphocytes and granulocytes (red arrowheads) were observed. After mono-therapy with 9-AMC, necrotic shedding of mucosal epithelial cells was seen in small intestinal tissues of mice (black arrows). Importantly, for the combination therapy of tigecycline+9-AMC (20 + 5 mg/kg), no obvious pathological changes were observed. Overall, the combination of tigecycline and 9-AMC can improve the pathological state of intestinal tissues to realize the potent therapeutic effects in mice.

## Discussions

The occurrence and increasing dissemination of tigecycline resistant genes *tet*(X4) dramatically compromise the efficacy of tigecycline as the last resort for bacterial infections, posing a severe threat for global public health (Markley and Wencewicz, 2018; Gasparrini et al., 2020; Cui et al., 2021, 2022; Rueda Furlan et al., 2023). Limited by the long and costly period to develop a novel drug, excavating the specific inhibitors or antimicrobial adjuvant to re-sensitive the activity of existed drugs against antimicrobial resistant bacteria have been regarded as effective strategies. Over the decades, the inhibitors of β-lactams such as the clavulanic acid, sulbactam, avibactam, etc., have been the typical success for the adjuvants identification to tackle antimicrobial resistance in clinical practice (Liras and Rodríguez-García, 2000; Barcelona et al., 2008; Ehmann et al., 2012; Yang et al., 2023). Till now, several tigecycline enhancers were discovered such as metformin (Liu et al., 2020a, 2020b; Xiao et al., 2022), bismuth nitrate Bi(NO_3_)_3_ (Deng et al., 2022), ML-7 (Sun et al., 2021). The antimicrobial potentiators restore the sensitivity by circumventing the intrinsic resistance mechanism or binding to the bacterial target proteins, altering the conformation of target proteins to inactivate the destroying enzymes, promoting the accumulation of tigecycline in bacterial cells, thereby reversing *tet*(X4)-mediated tigecycline resistance.

Metabolites, which share the same skeleton structure as the pro-drugs, are severely undervalued as potential antimicrobial adjuvants (Palmer et al., 2010; Wang et al., 2018). Many antimicrobials are chemically unstable and can be metabolized into more stable forms. As reported, antibiotic-resistant and sensitive strains may be affected by the short-term effects of the antibiotics and the potential long-term effects of its metabolites. Anhydrotetracycline is a key bio-synthetic precursor and degradation product of tetracycline (Markley et al., 2019; Jagdmann et al., 2022; Kumar et al., 2023). The degradation product is not degraded by tetracycline inactivating enzymes and can competitively block substrate binding to inactivating enzymes, restoring tetracycline sensitivity. Inspired by this, we put forward the hypothesis the potential of metabolites as the adjuvants.

The metabolic profile of tigecycline was conducted using ^14^C-labeled form (Hoffmann et al., 2007). Tigecycline distributed extensively partly metabolized and eliminated through feces and urine. The major metabolic pathways were glucuronidation and amide hydrolysis followed by *N*-acetylation. The identified metabolites of tigecycline include 9-aminominocycline, t-butylaminoacetic acid, tigecycline glucuronide, *N*-acetyl-9-aminominocycline, and hydroxylated tigecycline. Hopefully, we found that 9-AMC, one of tigecycline’s metabolites, exhibits synergistic antibacterial activity with tigecycline. The 9-AMC as a potentiator is unveiled before may due to the trace level.

In recent years, molecular docking has become an important technology in computer-aided drug development (Lin et al., 2023). The interactions and conformational relationships between antimicrobial adjuvants and Tet(X4)-inactivating enzymes can be simulated through molecular docking. The affinity capacity obtained by molecular docking indicated that azidothymidine showed high affinity and it localized the catalytic pocket, and inhibited the three Tet(X2/X3/X4)-inactivating enzymes (Liu et al., 2020a). Bismuth nitrate Bi(NO_3_)_3_ binds to the active center of the enzyme in a competitive binding manner, and the bismuth atom targets the Tet(X4) inactivating enzyme in a non-competitive manner, altering the structure of the major binding pocket and antagonizing the resistance of the resistant proteins to tigecycline (Deng et al., 2022). Plumbagin directly inhibited the enzyme-catalyzed pocket of Tet(X3/X4) (Xu et al., 2022). In the present study, 9-AMC can bind to the active center of Tet(X4) inactivating enzyme and interact with other active centers of FAD. In addition, the binding energy of 9-AMC to Tet(X4) inactivating enzyme was lower than that of tigecycline, suggesting that the binding of 9-AMC to the inactivating enzyme was more stable. The combination therapy of 9-AMC and tigecycline inhibited the growth of resistant bacteria, effectively blocked the evolution of the antimicrobial resistant gene *tet*(X4), and narrowed the mutation selection window, which was consistent with a recent published tigecycline adjuvant, saturated fatty acid. 9-AMC and tigecycline possesses antibacterial activity *in vitro* and *in vivo*, and safety evaluation studies verified no noticeable toxicity.

The further molecular mechanism research showed that 9-AMC increased the membrane permeability and promoted the oxidative damage. The obvious up-regulated expression of ROS often accompanied by down-regulated level of the intracellular ATP, leading to antimicrobial accumulation of tigecycline in *E.coli*. Additionally, ATP, as the most important energy molecule in biological organisms, plays an indispensable and important role in various physiological and pathological processes. Changes in the level of ATP will affect the function of cells. When bacteria are in apoptosis, necrosis, or in some toxic state, ATP levels decrease dramatically. In this experiment, we explored the effect on ATP synthesis level of drug-resistant bacteria. Bacterial membranes can generate the proton-motive force (PMF). PMF is the driving force for ATP synthesis via the electron transport chain, which is comprised of two components: mitochondrial membrane potential and H^+^ ion concentration gradient across the mitochondrial membrane (Cai et al., 2023). Dissipation of the PMF results in the loss of cell viability and even death. Herein, the fluorescent probes, DIOC2(3) and BCECF-AM were used to determine the membrane potential and trans-membrane proton gradient (ΔpH), respectively. The increased fluorescence intensity after treatment with 9-AMC and tigecycline reflected the disruption of membrane potential, which would be compensated by increasing ΔpH. Consistently, the up-regulation of fluorescence intensity in the combination therapy group increased compared to tigecycline monotherapy. The transcriptomics analysis demonstrated that 9-AMC damaged the membrane permeability and diminished the functions of efflux pump, thereby enhancing the antimicrobial activity.

## Conclusion

Overall, 9-AMC could effectively reverse the tigecycline resistance against the *tet*(X4)-positive *E.coli in vitro* and *in vivo*. 9-AMC inhibits the evolution of tigecycline resistance genes and narrows the mutant selection window. 9-AMC in combination with tigecycline could enhance membrane damage, reduce the intracellular ATP, and accelerate the oxidative damage, thereby restoring tigecycline activity. 9-AMC, one of tigecycline metabolites, is a promising enhancer to improve the therapeutic efficacy against tigecycline-resistant *tet*(X4)-positive *E.coli* infections.

## Data availability statement

The raw data supporting the conclusions of this article will be made available by the authors, without undue reservation.

## Ethics statement

All the animal experiments were approved by the Animal Welfare and Ethics Committee at Anhui Agricultural University with the approval ID of AHAUXMSQ2023052.

## Author contributions

FS: Conceptualization, Methodology, Writing – original draft. LZ: Formal analysis, Investigation, Methodology, Writing – original draft. XM: Software, Validation, Writing – original draft. TA: Validation, Writing – review & editing. YW: Funding acquisition, Supervision, Writing – review & editing. LL: Funding acquisition, Project administration, Writing – review & editing.

## References

[ref1] Barcelona L. Marin M. Stamboulian D. (2008). Betalactam antibiotics combined with bectalactamases inhibitors. Amoxicillin-sulbactam. Medicina 68, 65–74. 18416324

[ref2] Cai J. J. Shi J. R. Chen C. He M. P. Wang Z. Q. Liu Y. (2023). Structural-activity relationship-inspired the discovery of saturated fatty acids as novel colistin enhancers. Adv. Sci. 10:2302182. doi: 10.1002/advs.202302182, PMID: 37552809 PMC10582468

[ref3] Caneschi A. Bardhi A. Barbarossa A. Zaghini A. (2023). The use of antibiotics and antimicrobial resistance in veterinary medicine, a complex phenomenon: a narrative review. Antibiotics (Basel) 12:487. doi: 10.3390/antibiotics12030487, PMID: 36978354 PMC10044628

[ref4] Chen Y. Hu D. Zhang Q. Liao X. P. Liu Y. H. Sun J. (2017). Efflux pump overexpression contributes to tigecycline heteroresistance in *Salmonella enterica serovar Typhimurium*. Front. Cell. Infect. Microbiol. 7:37. doi: 10.3389/fcimb.2017.00037, PMID: 28261566 PMC5313504

[ref5] Cui C. Y. Chen Q. He Q. Chen C. Zhang R. M. Feng Y.. (2022). Transferability of tigecycline resistance: characterization of the expanding Tet(X) family. WIREs Mech. Dis. 14:e1538. doi: 10.1002/wsbm.1538, PMID: 35023325

[ref6] Cui C. Y. He Q. Jia Q. L. Li C. Chen C. Wu X. T.. (2021). Evolutionary trajectory of the Tet(X) family: critical residue changes towards high-level tigecycline resistance. mSystems 6. doi: 10.1128/mSystems.00050-21 PMC8269203 34006624

[ref7] Deng T. Jia Y. Tong Z. Shi J. Wang Z. Liu Y. (2022). Bismuth drugs reverse *tet*(X)-conferred tigecycline resistance in gram-negative bacteria. Microbiol. Spectr. 10:e0157821. doi: 10.1128/spectrum.01578-21, PMID: 35138168 PMC8826830

[ref8] Dhanda G. Acharya Y. Haldar J. (2023). Antibiotic adjuvants: a versatile approach to combat antibiotic resistance. ACS Omega 8, 10757–10783. doi: 10.1021/acsomega.3c00312, PMID: 37008128 PMC10061514

[ref9] Ehmann D. E. Jahic H. Ross P. L. Gu R. F. Hu J. Kern G.. (2012). Avibactam is a covalent, reversible, non-β-lactam β-lactamase inhibitor. Proc. Natl. Acad. Sci. USA 109, 11663–11668. doi: 10.1073/pnas.1205073109, PMID: 22753474 PMC3406822

[ref10] Forsberg K. J. Patel S. Wencewicz T. A. Dantas G. (2015). The tetracycline destructases: a novel family of tetracycline-inactivating enzymes. Chem. Biol. 22, 888–897. doi: 10.1016/j.chembiol.2015.05.017, PMID: 26097034 PMC4515146

[ref11] Gasparrini A. J. Markley J. L. Kumar H. Wang B. Fang L. Irum S.. (2020). Tetracycline-inactivating enzymes from environmental, human commensal, and pathogenic bacteria cause broad-spectrum tetracycline resistance. Commun. Biol. 3:241. doi: 10.1038/s42003-020-0966-5, PMID: 32415166 PMC7229144

[ref12] He T. Wang R. Liu D. Walsh T. R. Zhang R. Lv Y.. (2019). Emergence of plasmid-mediated high-level tigecycline resistance genes in animals and humans. Nat. Microbiol. 4, 1450–1456. doi: 10.1038/s41564-019-0445-2, PMID: 31133751

[ref13] Hoffmann M. DeMaio W. Jordan R. A. Talaat R. Harper D. Speth J.. (2007). Metabolism, excretion, and pharmacokinetics of [14C] tigecycline, a first-in-class glycylcycline antibiotic, after intravenous infusion to healthy male subjects. Drug Metab. Dispos. 35, 1543–1553. doi: 10.1124/dmd.107.015735, PMID: 17537869

[ref14] Hu Y. Zhang X. Deng S. Yue C. Jia X. Lyu Y. (2023). Non-antibiotic prevention and treatment against *Acinetobacter baumannii* infection: are vaccines and adjuvants effective strategies? Front. Microbiol. 14:1049917. doi: 10.3389/fmicb.2023.1049917, PMID: 36760499 PMC9905804

[ref15] Jagdmann J. Andersson D. I. Nicoloff H. (2022). Low levels of tetracyclines select for a mutation that prevents the evolution of high-level resistance to tigecycline. PLoS Biol. 20:e3001808. doi: 10.1371/journal.pbio.3001808, PMID: 36170241 PMC9550176

[ref16] Kumar H. Williford E. E. Blake K. S. Virgin-Downey B. Dantas G. Wencewicz T. A.. (2023). Structure of anhydrotetracycline-bound Tet(X6) reveals the mechanism for inhibition of type 1 tetracycline destructases. Commun. Biol. 6:423. doi: 10.1038/s42003-023-04792-4, PMID: 37062778 PMC10106456

[ref17] Li R. Chen X. Zhou C. Dai Q. Q. Yang L. (2022). Recent advances in beta-lactamase inhibitor chemotypes and inhibition modes. Eur. J. Med. Chem. 242:114677. doi: 10.1016/j.ejmech.2022.114677, PMID: 35988449

[ref18] Li Y. Wang Q. Peng K. Liu Y. Xiao X. Mohsin M.. (2021). Distribution and genomic characterization of tigecycline-resistant *tet*(X4)-positive *Escherichia coli* of swine farm origin. Microb. Genom. 7:000667. doi: 10.1099/mgen.0.000667, PMID: 34693904 PMC8627205

[ref19] Lin R. K. Zhang J. H. Xu R. X. Yuan C. Guo L. Liu P. F.. (2023). Developments in molecular docking technologies for application of polysaccharide-based materials: a review. Crit. Rev. Food Sci., 1–13. doi: 10.1080/10408398.2023.2200833, PMID: 37077154

[ref20] Liras P. Rodríguez-García A. (2000). Clavulanic acid, a β-lactamase inhibitor: biosynthesis and molecular genetics. Appl. Microbiol. Biotechnol. 54, 467–475. doi: 10.1007/s002530000420 11092620

[ref21] Liu Y. Jia Y. Yang K. Li R. Xiao X. Wang Z. (2020a). Anti-HIV agent azidothymidine decreases Tet(X)-mediated bacterial resistance to tigecycline in *Escherichia coli*. Commun. Biol. 3:162. doi: 10.1038/s42003-020-0877-5, PMID: 32246108 PMC7125129

[ref22] Liu Y. Jia Y. Yang K. Li R. Xiao X. Zhu K.. (2020b). Metformin restores tetracyclines susceptibility against multidrug resistant bacteria. Adv. Sci. (Weinh) 7:1902227. doi: 10.1002/advs.201902227, PMID: 32596101 PMC7312304

[ref23] Markley J. L. Fang L. Gasparrini A. J. Symister C. T. Kumar H. Tolia N. H.. (2019). Semisynthetic analogues of anhydrotetracycline as inhibitors of tetracycline destructase enzymes. ACS Infect. Dis. 5, 618–633. doi: 10.1021/acsinfecdis.8b00349, PMID: 30835428 PMC6490184

[ref24] Markley J. L. Wencewicz T. A. (2018). Tetracycline-inactivating enzymes. Front. Microbiol. 9:9. doi: 10.3389/fmicb.2018.01058 29899733 PMC5988894

[ref25] Mohsin M. Hassan B. Martins W. Li R. Abdullah S. Sands K.. (2021). Emergence of plasmid-mediated tigecycline resistance *tet*(X4) gene in *Escherichia coli* isolated from poultry, food and the environment in South Asia. Sci. Total Environ. 787:147613. doi: 10.1016/j.scitotenv.2021.147613, PMID: 33992939

[ref26] Palmer A. C. Angelino E. Kishony R. (2010). Chemical decay of an antibiotic inverts selection for resistance. Nat. Chem. Biol. 6, 105–107. doi: 10.1038/Nchembio.289, PMID: 20081825 PMC2811317

[ref27] Peterson L. R. (2008). A review of tigecycline – the first glycylcycline. Int. J. Antimicrob. Agents 32, S215–S222. doi: 10.1016/S0924-8579(09)70005-6, PMID: 19134522

[ref28] Ren Z. Yang S. Han J. Nie C. Wang C. Wang J.. (2023). Reduction of antibiotic use and multi-drug resistance bacteria infection in neonates after improvement of antibiotics use strategy in a level 4 neonatal intensive care unit in southern China. Eur. J. Clin. Microbiol. Infect. Dis. 42, 87–98. doi: 10.1007/s10096-022-04522-4, PMID: 36409375

[ref29] Ruan Z. Jia H. Chen H. Wu J. He F. Feng Y. (2020). Co-existence of plasmid-mediated tigecycline and colistin resistance genes *tet*(X4) and mcr-1 in a community-acquired *Escherichia coli* isolate in China. J. Antimicrob. Chemother. 75, 3400–3402. doi: 10.1093/jac/dkaa317, PMID: 32747957

[ref30] Rueda Furlan J. P. Fuentes-Castillo D. Guedes Stehling E. Lincopan N. Sellera F. P. (2023). The emergence of tet(X) variants highlight challenges for the global genomic surveillance of tigecycline resistance. Lancet Microbe 4:e857. doi: 10.1016/S2666-5247(23)00249-5, PMID: 37634526

[ref31] Sodeifian F. Zangiabadian M. Arabpour E. Kian N. Yazarlou F. Goudarzi M.. (2023). Tigecycline-containing regimens and multi drug-resistant *Acinetobacter baumannii*: a systematic review and meta-analysis. Microb. Drug Resist. 29, 344–359. doi: 10.1089/mdr.2022.0248, PMID: 37192494

[ref32] Sun L. Sun L. Li X. Hu X. Wang X. Nie T.. (2021). A novel tigecycline adjuvant ML-7 reverses the susceptibility of tigecycline-resistant *Klebsiella pneumoniae*. Front. Cell. Infect. Microbiol. 11:809542. doi: 10.3389/fcimb.2021.809542, PMID: 35071055 PMC8766836

[ref33] Ueda T. Takesue Y. Nakajima K. Ichiki K. Ishikawa K. Yamada K.. (2023). Correlation between antimicrobial resistance and the hospital-wide diverse use of broad-spectrum antibiotics by the antimicrobial stewardship program in Japan. Pharmaceutics 15:518. doi: 10.3390/pharmaceutics15020518, PMID: 36839839 PMC9964530

[ref34] Wang Y. Liu F. Zhu B. Gao G. F. (2020). Discovery of tigecycline resistance genes *tet*(X3) and *tet*(X4) in live poultry market worker gut microbiomes and the surrounded environment. Sci. Bull. 65, 340–342. doi: 10.1016/j.scib.2019.12.027, PMID: 36659223

[ref35] Wang J. Wu H. Mei C. Y. Wang Y. Wang Z. Y. Lu M. J.. (2021). Multiple mechanisms of tigecycline resistance in *Enterobacteriaceae* from a pig farm, China. Microbiol. Spectr. 9:e0041621. doi: 10.1128/Spectrum.00416-21, PMID: 34523976 PMC8557919

[ref36] Wang Y. L. Zhou Y. Zheng Z. N. Li J. T. Yan Y. T. Wu W. (2018). Sulforaphane metabolites reduce resistance to paclitaxel via microtubule disruption. Cell Death Dis. 9:1134. doi: 10.1038/s41419-018-1174-9, PMID: 30429459 PMC6235886

[ref37] Xiao X. Huan Q. Huang Y. Liu Y. Li R. Xu X.. (2022). Metformin reverses tmexCD1-toprJ1- and *tet*(a)-mediated high-level tigecycline resistance in *K. pneumoniae*. Antibiotics (Basel) 11:162. doi: 10.3390/antibiotics11020162 35203765 PMC8868462

[ref38] Xu L. Zhou Y. Niu S. Liu Z. Zou Y. Yang Y.. (2022). A novel inhibitor of monooxygenase reversed the activity of tetracyclines against tet(X3)/tet(X4)-positive bacteria. EBioMedicine 78:103943. doi: 10.1016/j.ebiom.2022.103943, PMID: 35306337 PMC8933826

[ref39] Yang Y. Yan Y. H. Schofield C. J. McNally A. Zong Z. Li G. B. (2023). Metallo-beta-lactamase-mediated antimicrobial resistance and progress in inhibitor discovery. Trends Microbiol. 31, 735–748. doi: 10.1016/j.tim.2023.01.013, PMID: 36858862

[ref40] Zheng E. J. Andrews I. W. Grote A. T. Manson A. L. Alcantar M. A. Earl A. M.. (2022). Modulating the evolutionary trajectory of tolerance using antibiotics with different metabolic dependencies. Nat. Commun. 13:2525. doi: 10.1038/s41467-022-30272-0, PMID: 35534481 PMC9085803

