# Supplementary material for: A promising metabolite, 9-aminominocycline, restores the sensitivity of tigecycline against tet(X4)-positive Escherichia coli
Source: Front Microbiol. 2024 Jul 8;15:1432320. doi: 10.3389/fmicb.2024.1432320 (PMC11264248; doi:10.3389/fmicb.2024.1432320)
Supplement: Supplementary file 1 [file Table_1.DOCX]

**Supplementary**

**Supplementary Tables**

Supplementary Tables. S1 The strain information of *tet*(X4)-positive *E.coli*

| Name | Source | Time | Remarks |
| --- | --- | --- | --- |
| *E.coli* 47R | Pig farms in Qingdao | 2018 | from Professor Wang Yang, China Agricultural University |
| *E.coli* 2DZ50T | Pig slaughterhouses in Qingdao | 2019 |  |
| *E.coli* DH5α-pET30a+*tet*(X4) | our laboratory | 2022 | Reconstructed in our lab |

Supplementary Tables. S2 The primer information of *tet*(X4)

| Gene | Primer sequences(5'-3') |
| --- | --- |
| *tet*(X4) | F-CGCCATATGCATGAGCAATAAAGAAAAACAAATGA |
|  | R-CCCAAGCTTTTATACATTTAACAATTGCTGAAAC |

Supplementary Tables. S3 Fluorescent quantitative PCR amplification primers

| Gene | Primer sequences(5'-3') | Temperature/℃ | Length/bp |
| --- | --- | --- | --- |
| 16S rRNA | F-TTGAGTCTCGTAGAGGGGGGTA | 60 | 194 |
|  | R-TCAAGGGCACAACCTCCAAGT |  |  |
| *acrZ* | F-TGGTATTCGCCGTAATCATGGTA | 60 | 122 |
|  | R-ATTTTGTCCGGGCTGGTCTT |  |  |
| *mdtM* | F-GCAATGATGGCAAAAAGGACT | 60 | 157 |
|  | R-GTTATGTCACGGTGCAGGAGG |  |  |
| *ompW* | F-TAACGATCATGGCAAAGAGGC | 60 | 92 |
|  | R-TAATCAGATAATCAACCCCCACC |  |  |
| *nikA* | F-CACCCTGTTTGCCCCTTCT | 60 | 145 |
|  | R-CGGCTGACCATTTTTCTCG |  |  |
| *artJ* | F-ATTCAGTTTTTCCAGCAGGGC | 60 | 169 |
|  | R-TGGTCGTATTGATGGGGTATTTG |  |  |

Supplementary Tables. S4 Molecular docking of TGC and 9-AMC with Tet(X4) protein

| Molecule | Docking site | Docking mode | Dond length | Binding energy（Kcal/mol） |
| --- | --- | --- | --- | --- |
| TGC | ASP-311 | hydrogen bond | 3.4 | -6.23 |
|  | ARG-117 |  | 3.1 |  |
| 9-AMC | ASN-226 |  | 3.6 | -8.27 |
|  | THR-59 |  | 3.1 |  |
|  | GLY-58 |  | 3.0 |  |

Supplementary Tables. S5 Mouse blood routine indicators

| Parameters | Unit | Control | TGC | TGC+9-AMC |
| --- | --- | --- | --- | --- |
| WBC | 10^9/L | 5.82±0.37 | 5.93±0.48 | 6.06±0.65 |
| NEU | % | 18.87±2.52 | 18.00±2.71 | 19.40±1.20 |
| LYM | % | 74.43±4.58 | 75.23±3.65 | 75.30±1.55 |
| MON | % | 5.53±2.29 | 5.80±1.16 | 3.70±1.06 |
| EOS | % | 0.97±0.12 | 0.97±0.29 | 1.53±0.56 |
| BAS | % | 0.20±0.16 | 0 | 0.07±0.05 |
| RBC | 10^12/L | 8.83±0.31 | 9.04±0.67 | 8.26±0.39 |
| HGB | g/L | 149.67±8.26 | 151.00±5.66 | 136.00±1.41 |
| HCT | % | 44.73±1.84 | 44.93±2.00 | 40.47±0.97 |
| MCV | fL | 50.67±0.87 | 49.80±2.06 | 49.00±1.14 |
| MCH | pg | 16.93±0.39 | 16.70±0.62 | 16.53±0.62 |
| MCHC | g/L | 334.00±7.79 | 335.67±5.25 | 336.33±4.78 |
| RDW-CV | % | 12.77±0.56 | 13.27±0.80 | 12.90±0.73 |
| RDW-SD | fL | 27.27±0.93 | 27.90±2.70 | 26.47±1.03 |
| PLT | 10^9/L | 748.33±131.31 | 888.67±147.88 | 643.00±42.06 |
| MPV | fL | 5.77±0.33 | 5.93±0.31 | 5.87±0.12 |
| PDW |  | 15.50±0.08 | 15.60±0.08 | 15.43±0.12 |
| PCT | % | 0.43±0.07 | 0.52±0.06 | 0.38±0.03 |

Supplementary Tables. S6 Biochemical indicators of mice

| Parameters | Unit | Control | TGC | TGC+9-AMC |
| --- | --- | --- | --- | --- |
| Ca^2+^ | mmol/L | 2.36±0.06 | 2.49±0.04 | 2.35±0.09 |
| Glu-G | mmol/L | 8.72±0.65 | 9.67±0.70 | 10.72±1.21 |
| P Ⅱ | mmol/L | 2.97±0.31 | 3.57±0.31 | 3.23±0.43 |
| ALT | U/L | 41.10±2.27 | 44.57±4.23 | 37.87±4.24 |
| AST | U/L | 148.13±26.88 | 188.70±48.41 | 129.93±7.74 |
| ALP | U/L | 262.47±18.80 | 328.17±72.33 | 251.47±10.98 |
| TG | mmol/L | 1.84±0.86 | 1.96±0.19 | 1.89±0.34 |
| TP Ⅱ | g/L | 52.83±2.64 | 55.23±1.56 | 50.97±1.73 |
| ALB Ⅱ | g/L | 32.00±2.38 | 35.60±0.50 | 31.47±1.54 |
| TC | mmol/L | 2.16±0.15 | 2.19±0.39 | 2.33±0.38 |
| CREA-S | μmol/L | 19.63±0.41 | 19.70±1.43 | 20.37±1.23 |
| UREA | mmol/L | 9.10±0.68 | 8.32±1.35 | 8.21±0.67 |

Supplementary Tables. S7 Evaluation of pathological changes in the small intestine of mice

| Pathology  Group | Erosion | Inflammatory infiltration | Separation of mucosal epithelium from lamina propria |
| --- | --- | --- | --- |
| Normal | 0 | 0 | 0 |
| PBS | 2 | 1 | 0 |
| TGC | 1 | 1 | 0 |
| 9-AMC | 1 | 0 | 0 |
| Combination | 0 | 0 | 0 |

**Supplementary Figures**


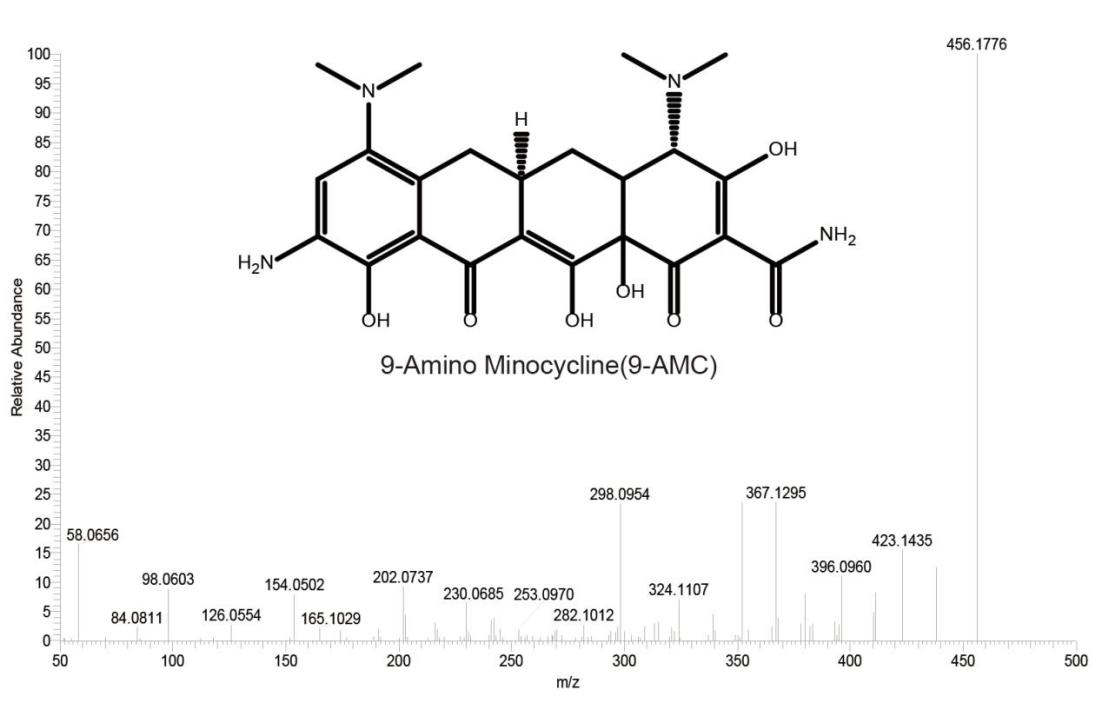


Figure S1. The MS/MS spectrogram and structural formula of 9-AMC.


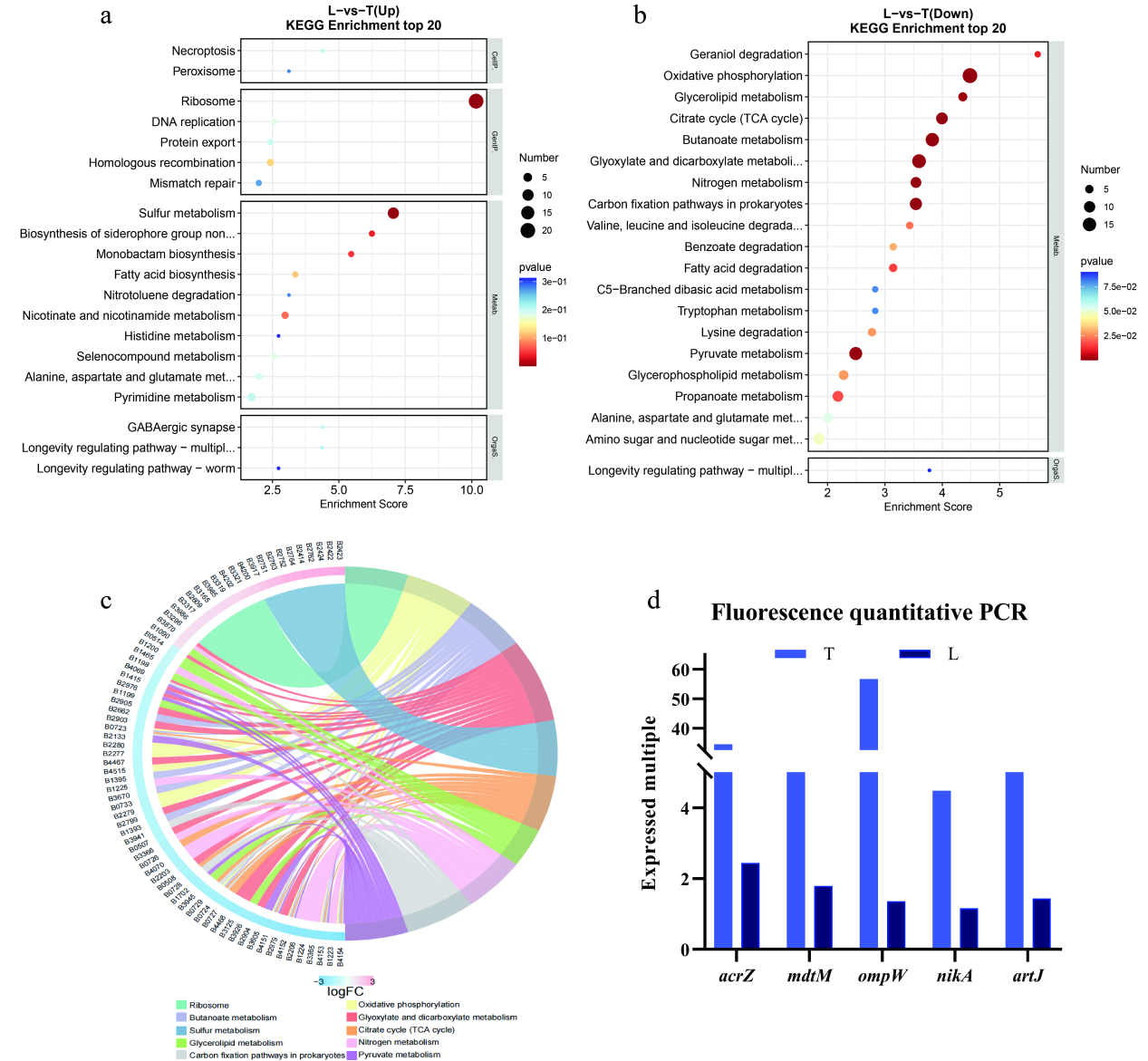


Figure S2. The results of transcriptome and fluorescence quantitative PCR


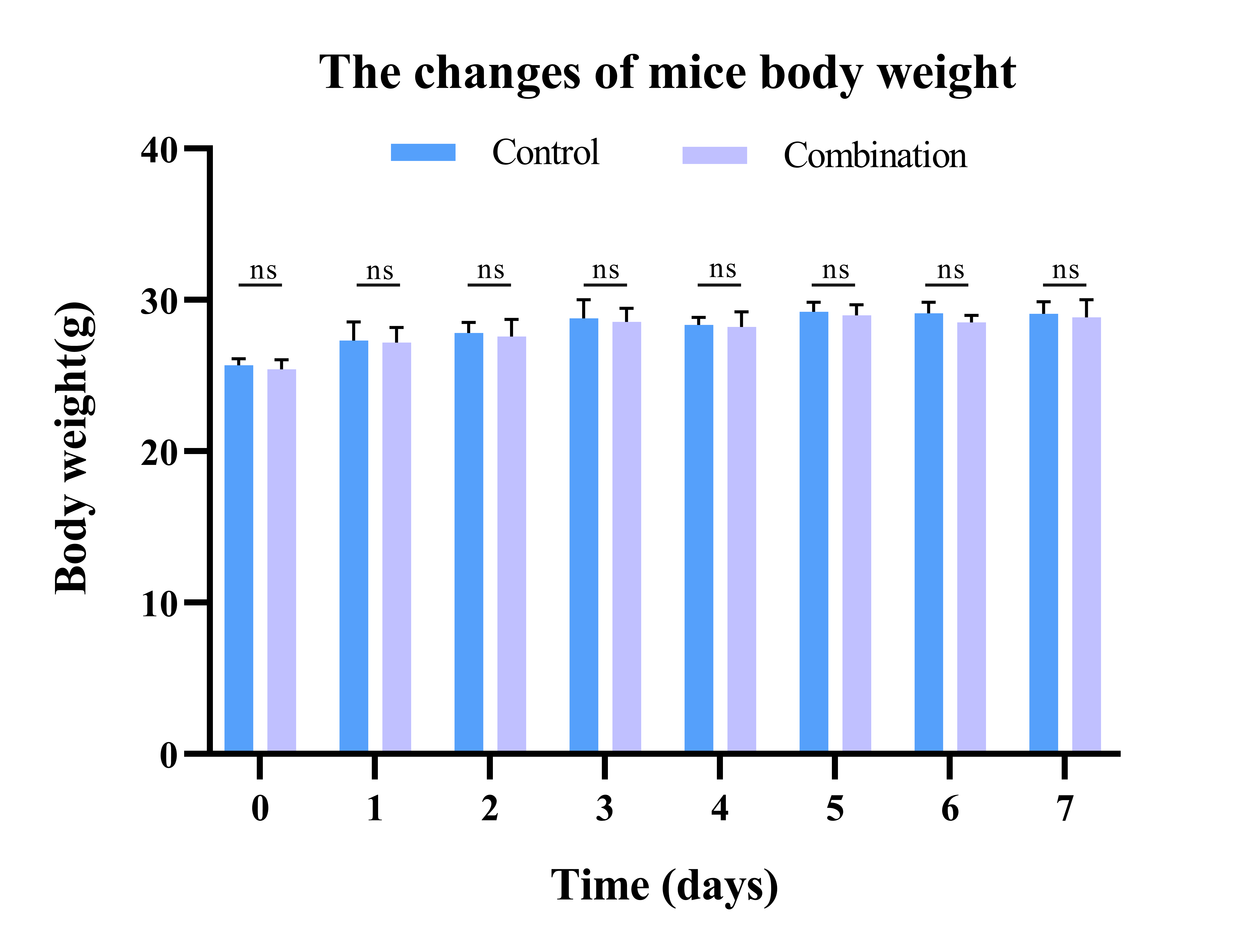


Figure S3. Body weight changes in mice within seven days after combination of tigecycline and 9-AMC.

Seven day changes in body weight of healthy mice and mice treated with high-dose medication, the combination of tigecycline (20 mg/kg) and 9-AMC (128 mg/kg) (*n* = 6 per group). One-way ANOVA was used to determine statistical significance (ns p > 0.05).
